# Large Eddy Simulation and Thermodynamic Design of the Organic Rankine Cycle Based on Butane Working Fluid and the High-Boiling-Point Phenyl Naphthalene Liquid Heating System

**DOI:** 10.3390/e24101461

**Published:** 2022-10-13

**Authors:** Alon Davidy

**Affiliations:** Independent Research Scientist, Petach Tiqwa 4942136, Israel; alon.davidy@gmail.com; Tel.: +972-03-9049118

**Keywords:** ORC, THERMOPTIM thermodynamic software, petroleum coke, phenyl naphthalene liquid

## Abstract

Large Eddy Simulation (LES) and Thermodynamic study have been performed on Organic Rankine Cycle (ORC) components (boiler, evaporator, turbine, pump, and condenser). The petroleum Coke burner provided the heat flux needed for the butane evaporator. High boiling point fluid (called phenyl-naphthalene) has been applied in the ORC. The high boiling liquid is safer (steam explosion hazard may be prevented) for heating the butane stream. It has best exergy efficiency. It is non-corrosive, highly stable, and flammable. Fire Dynamics Simulator software (FDS) has been applied in order to simulate the pet-coke combustion and calculate the Heat Release Rate (HRR). The maximal temperature of the 2-Phenylnaphthalene flowing in the boiler is much less than its boiling temperature (600 K). Enthalpy, entropy and specific volume required for evaluating the heat rates and the power have been computed by employing the THERMOPTIM thermodynamic code. The proposed design ORC is safer. This is because the flammable butane is separated from the flame produced in the petroleum coke burner. The proposed ORC obeys the two fundamental laws of thermodynamics. The calculated net power is 3260 kW. It is in good agreement with net power is reported in the literature. The thermal efficiency of the ORC is 18.0%.

## 1. Introduction

Heavy industries (such as refineries, cement factories, and coal power plants) are considered sources of carbon dioxide emissions. One of the technologies being developed to aid in the process of carbon reduction is called the Organic Rankine Cycle (ORC). This system is applied for power production from low to high medium-temperature heat sources. ORC systems apply organic fluids instead of water in order to generate electricity. Because of their lower boiling points and higher vapor pressure compared to water, lower-temperature heat sources can be utilized in order to generate electricity more efficiently. This technology may reduce the carbon emissions footprint and operational costs and increase the flexibility [1]. The ORC has been successfully applied for use in geothermal power generation, solar thermal power production, waste heat recovery, and biomass. This technology has reached a considerable degree of maturity [2]. Several research studies dealing with the utilization of pet coke for power production have been conducted. Hamadeh et al. [3] carried out a technical and economic analysis of an oxy-petroleum coke plant with CO_2_ capture by applying Aspen PlusTM software 8.8 (20 Crosby Drive, Bedford, Massachusetts 01730, USA). Shen et al. [4] performed a feasibility economic study of Coal to Liquids (CTL) systems. The Fischer–Tropsch (F-T) subsystem was incorporated into an IGCC (integrated gasification combined cycle) plant to co-produce electric power, CTL fuels, chemicals, and steam. The Heat Recovery Steam Generator may be applied in order to produce electricity. Méndez-Cruz et al. performed a thermodynamic analysis of the performance of an ORC by applying four different working fluids: R134a, R600a, R245fa, and R123 [5]. Comparison of the exergy viability index for the case studies indicated that R245fa and R600a are the most viable working fluids in the ORC operation. Fernández Guillamón et al. optimized the performance of the ORC super-heater pressure [6]. They showed that R123 may replace R236EA and R245FA. The working fluids R1234ZE and R1234YF behave similarly to R134A. The maximal thermal efficiency of the ORC may reach 23–25%. This paper proposes a new method to supply heat to the Organic Rankine Cycle. It is composed of CFD modelling of pet-coke combustion and a thermodynamic analysis of the ORC. The proposed design is safer. This is because the flammable butane is separated from the flame produced in the petroleum coke burner. A new bio-economic indicator was applied in this study. This indicator allows us to compare the power cycles (such as Rankine and Brayton) in relation to their sustainability. This indicator decreases as the thermal efficiency increases. This ORC power plant can be near to Delayed Coker Unit (DCU) which produces petroleum coke. Green hydrogen may be produced by using water splitting method. The proposed ORC is a modular system; the heating system (which includes the burner and the boiler) may be connected to the evaporator instead of the existing heating system. It may be also utilized for reinforcing other ORC power cycles (such as geothermal or solar) or for marine propulsion systems. It may be utilized for combined biodiesel (FAME) or γ-Valerolactone (GVL) and power productions. The structure of this paper is as follows: In Section 1.1, the ORC is described. In Section 1.2, the advantages of applying high-boiling liquids are presented. The fire dynamics simulator (FDS) software is described in Section 2.1 and Section 2.2. Section 2.3 describes the thermodynamic analysis of the organic Rankine cycle. Section 2.4 describes the thermo-physical properties of 2-phenylnaphthalene (C_16_H_12_). The thermodynamic analysis of the propane is described in Section 2.5. Section 3 presents the FDS models and the thermodynamic results.

### 1.1. The Organic Rankine Cycle

**Figure 1:**
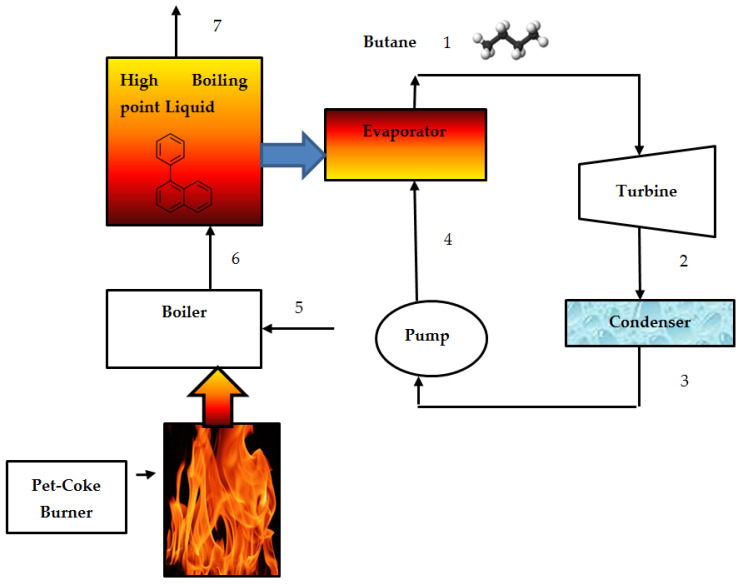
Schematics of the ORC system based on high-boiling-point liquid.

Butane was selected as the working fluid. Butane was pumped into the evaporator. High-boiling-point phenyl-naphthalene liquid was used to heat the butane in the evaporator. It was evaporated and then expanded in the turbine, generating mechanical shaft power. After expansion to a low pressure, the butane vapor was condensed in an air-cooled condenser. The butane liquid was then pumped to complete the thermodynamic cycle. The ORC system is shown in Figure 1.

### 1.2. Advantages of Applying High-Boiling Liquids

These liquids were applied for high temperature heating. These liquids were applied because of several reasons [7,8]:

(1) They operate reliably under atmospheric pressure;
(2) The heating temperature can be varied easily;
(3) They are safer to use (steam explosion hazard may be prevented);
(4) Heavy forgings for pressure vessels and piping are eliminated;
(5) They are compatible with low-cost materials (plain carbon steel and aluminum alloys can be applied) and have no corrosion potential.

#### Boiling Regimes of Water

**Figure 2:**
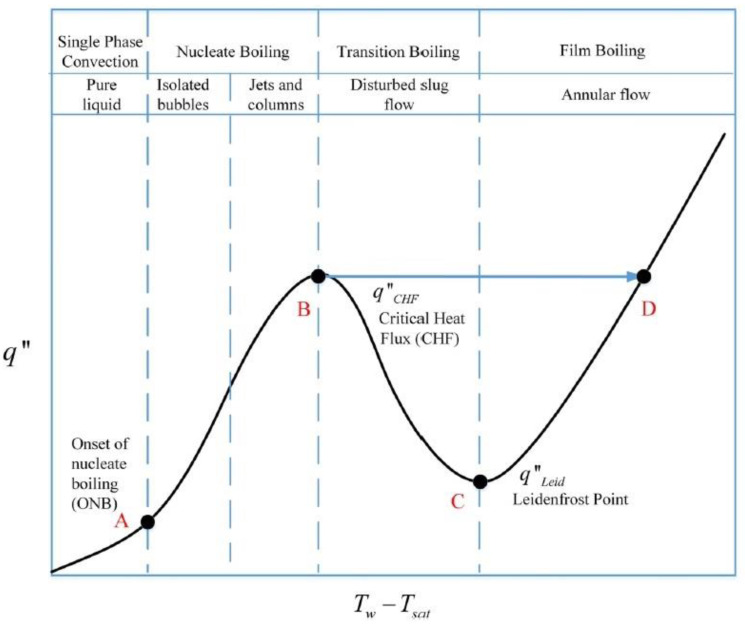
Boiling curve of water [10].

All major power plants depend upon the evaporation of water in boilers and the subsequent condensation of the vapor in condensers. The production of liquid fuel in oil refineries depends upon evaporation and condensation in fractional distillation columns. Thus, it is evident that two-phase flow is an integral part of the majority of processes upon which our industrial society is based [9]. Figure 2 shows the typical boiling curve of water [10].

This curve is composed of four segments. The first segment represents the single-phase convection regime. The OA line is almost straight (The slope is equal to the convective coefficient). The second segment represents the nucleate boiling regime (it is divided into two regimes: isolated bubbles and Jets and columns). The slope of the AB line is greater than that of the OA line. The AB line terminates at the point B (critical heat flux). The third segment represents the boiling transition. The heat flux (segment BC) decreases as the temperature drop rises and reaches a minimum at point C. It is called the Leiden-frost point. The last segment (line CD) is called film boiling (or annular flow). In this segment, the flux increases again with the temperature gradient (ΔT) [11]. In order to enhance the heat transfer and to increase the boiling point of the water, the saturation pressure of the water should be increased. By using high boiling point liquid, heavy forgings for pressure vessels and piping are eliminated. They are compatible with low-cost materials (plain carbon steel and aluminum alloys can be applied) and have no corrosion potential.

## 2. Materials and Methods

### 2.1. Fire Dynamic Simulation (FDS) Modeling of the Pet-Coke Burner

The 3D numerical simulation model of the combustion plant includes three models:

**Hydrodynamic Model**: It is solved by using the Finite Volume Method (FVM). The turbulence is modeled by using Large Eddy Simulation (LES). FDS is suitable for low Mach numbers.

**Combustion Model**: This model consists of two models. The first (default) uses the mixture fraction. Arrhenius reactions coefficients are applied for the second model.

**Radiation Transport**: The FVM method is applied for solving the radiation transport equation (RTE). The calculated temperatures, velocity, and products mole fractions are shown by using “smoke-view” postprocessor software. The transport equations of the FDS software are described in detail in [12,13,14,15].

### 2.2. FDS Modelling of the Combustor

**Figure 3:**
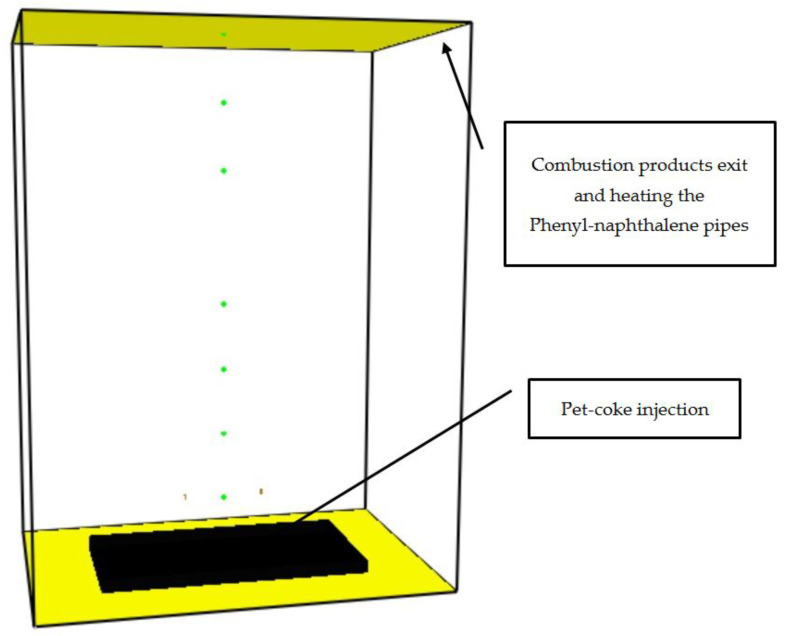
Geometrical model of the pet-coke burner [16].

The geometric model of the petroleum coke burner is shown in Figure 3 [16].

The burner height was 4.0 m. Its length and width were 4.0, 2.0, and 3.0 m, respectively. The coke particles were injected and ignited at the bottom side of the burner. Thermocouples and CO2 concentration sensors were positioned in several locations inside the model. Details on the positions of the thermocouples and the sensors are described in [16]. The pet-coke heat of combustion is 38,379 (kJ/kg) [17].

**Initial and Boundary conditions:** The burner compartment walls were opened. The initial temperature, the component concentration in the air, and the pressure were

$$T_{0}=20\;{}^{\circ}C\;;\;c_{O_{2},0}=0.21;c_{N_{2},0}=0.79;\;c_{i,0}=0\;;\;p=101,325\;\mathrm{Pa}$$

The results obtained in this work were compared to experimental and theoretical results obtained in [18]. The mass flow rate of the injected pet-coke particles is 0.84 kg/s [18]. Air enters the burner at a temperature of 20 °C [18].

### 2.3. Thermodynamic Analysis of the Organic Rankine Cycle

The Steady State Steady Flow (SSSF) process was considered. According to [18], the mass conservation equation is

(1) $$\sum {\dot{m}}_{i}=\sum {\dot{m}}_{e}$$

where ${\dot{m}}_{i}$ and ${\dot{m}}_{e}$ represent the mass flow rates entering the ORC component (such as evporator, turbine, condenser, and pump) and leaving the component. The first law of thermodynmics for the SSSF process is shown in the following equation [19]:

(2) $${\dot{Q}}_{c.v}+\sum {\dot{m}}_{i}\left(h_{i}+\frac{V_{i}^{2}}{2}+gZ_{i}\right)=\sum {\dot{m}}_{e}\left(h_{e}+\frac{V_{e}^{2}}{2}+gZ_{e}\right)+{\dot{W}}_{c.v}$$

where ${\dot{Q}}_{c.v}$ is the heat power produced/invested in the ORC component. ${\dot{W}}_{c.v}$ is the power invested/produced in the control volume. $h_{i}$ is the entalphy of the incoming fluid. $h_{e}$ is the entalphy of the leaving fluid. $V_{i}$ and $Z_{i}$ are the velocity and height of the incoming fluids, resepectively. $V_{e}$ and $Z_{e}$ are the velocity and height of the fluids leaving the component, resepectively. The second law of thermodynamics for the Steady State Steady Flow process is shown in the following equation [19]:

(3) $${\dot{S}}_{net}=\frac{dS_{net}}{dt}=\frac{dS_{c.v}}{dt}+\frac{dS_{surr}}{dt}=-\sum \frac{{\dot{Q}}_{c.v}}{T_{R}}+\sum {\dot{m}}_{e}s_{e}-\sum {\dot{m}}_{i}s_{i}\geq 0$$

where $s_{i}$ and $s_{e}$ represent the entropy of the incoming fluid and leaving fluid. t represents the time and $T_{R}$ denotes the reservoir high tempearture (or pet-coke flame temperarture). Butane flows inside the ORC cycle. The entropy, enthalpy, and specific volume properties of butane required to evaluate the heating rate and power were computed by employing the THERMOPTIM code [20]. The phenyl-naphthalene temperature at the boiler outlet (see Figure 1) was computed by applying the first law of thermodynamics:

(4) $${\dot{m}}_{phenyl}h_{6}={\dot{m}}_{phenyl}h_{5}+{\dot{Q}}_{HRR}$$

where ${\dot{Q}}_{HRR}$ is the average heat release rate inside the burner in [kW]. This term was calculated using the FDS software (see Figure 9). The phenylnaphthalene temperature at the evaporator outlet (see Figure 1) was computed by employing the energy balance equation on the heat evaporator:

(5) $$T_{7}=T_{6}-\frac{{\dot{m}}_{but}\left(h_{1}-h_{4}\right)}{{\dot{m}}_{phenyl}\cdot c_{p,phenyl}}$$

It was assumed the mass flow rate of phenyl-naphthalene is 52.6 kg/s. The enthalpy of butane at the outlet of the turbine was calculated by using Equation (6)

(6) $$\varepsilon_{t}=\frac{h_{1}-h_{2}}{h_{1}-h_{2s}}$$

where *ε_t_* is the isentropic efficiency of the turbine. The isentropic efficiency of the turbine (expander) was assumed to be 0.96. The enthalpy of *h*_2*s*_ was evaluated by assuming that *s*_1_ = *s*_2*s*_ (isentropic expansion) and *p*_2_ = 238 kPa. The rate of heat removed in the condenser was calculated by using the following equation:

(7) $${\dot{m}}_{But}h_{2}+{\dot{Q}}_{cond}={\dot{m}}_{But}h_{3}$$

It was assumed that the liquid butane exited from the condenser. The enthalpy of the butame at the outlet of the pump was calculated by using Equation (8):

(8) $$\varepsilon_{p}=\frac{h_{3}-h_{4s}}{h_{3}-h_{4}}=\frac{v_{3}\left(p_{3}-p_{4}\right)}{h_{3}-h_{4}}$$

The isentropic efficiency of the pump was assumed to be 0.94. The thermodynamic properties of 2-Phenylnaphthalene at the inlets and the outlets of the boiler and evaporator are shown in Section 2.4.

### 2.4. Calculation of Net Entropy Change Rate of the O

**Figure 4:**
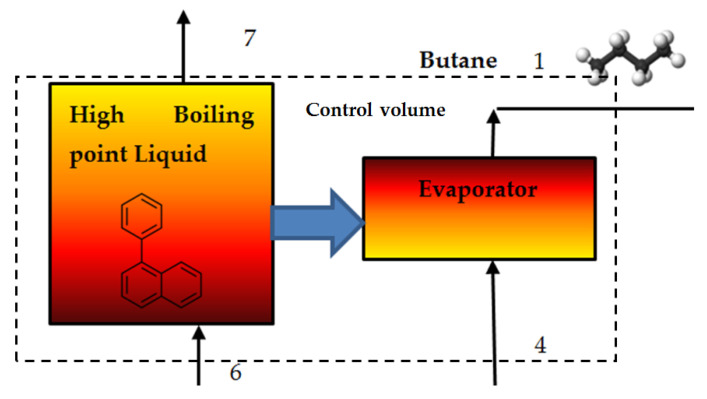
Definition of the evaporator control volume boundaries.

In the framework of this research work, I have applied the second law of the thermodynamics for control volume (see Figure 4).

I have defined the butane evaporator as the control volume. The overall entropy rate of the composed of the entropy rate of the control volume and the surroundings entropy rate (see Equation (3)).

For the steady state, steady flow process, we conclude that there no change with time of the entropy rate at any point within the control volume, and therefore, the first term of Equation (3) equals zero. That is

(9) $$\frac{dS_{c.v}}{dt}=0$$

Thus, for the SSSF process, the following equation is obtained:

(10) $$\frac{dS_{net}}{dt}=\frac{dS_{surr}}{dt}=-\sum \frac{{\dot{Q}}_{c.v}}{T_{R}}+\sum {\dot{m}}_{e}s_{e}-\sum {\dot{m}}_{i}s_{i}\geq 0$$

It should be noted that there is not heat interaction between the boundaries of the control volume (heat is transferred inside the control volume, i.e., between the hot Phenyl naphthalene stream and the cold butane stream). Thus [19]:

(11) $$\frac{dS_{net}}{dt}=\frac{dS_{surr}}{dt}=\sum {\dot{m}}_{e}s_{e}-\sum {\dot{m}}_{i}s_{i}\geq 0$$

The net entropy change rate of the evaporator is:

(12) $$\frac{dS_{E}}{dt}={\dot{m}}_{phenyl}s_{7}+{\dot{m}}_{But}s_{1}-{\dot{m}}_{phenyl}s_{6}-{\dot{m}}_{But}s_{4}$$

The net entropy change rate of the turbine is:

(13) $$\frac{dS_{turb}}{dt}={\dot{m}}_{But}s_{2}-{\dot{m}}_{But}s_{1}$$

The net entropy change rate of the condenser is:

(14) $$\frac{dS_{cond}}{dt}={\dot{m}}_{But}s_{3}-{\dot{m}}_{But}s_{2}-\frac{Q_{cond}}{T_{sat}\left(p_{3}\right)}$$

The net entropy change rate of the condenser is:

(15) $$\frac{dS_{pump}}{dt}={\dot{m}}_{But}s_{4}-{\dot{m}}_{But}s_{3}$$

### 2.5. Thermo-Physical and Thermodynamic Properties of 2-Phenylnaphthalene (C_16_H_12_)

The thermo-physical properties of 2-phenylnaphthalene are provided in Table 1 [21].

**Figure 5:**
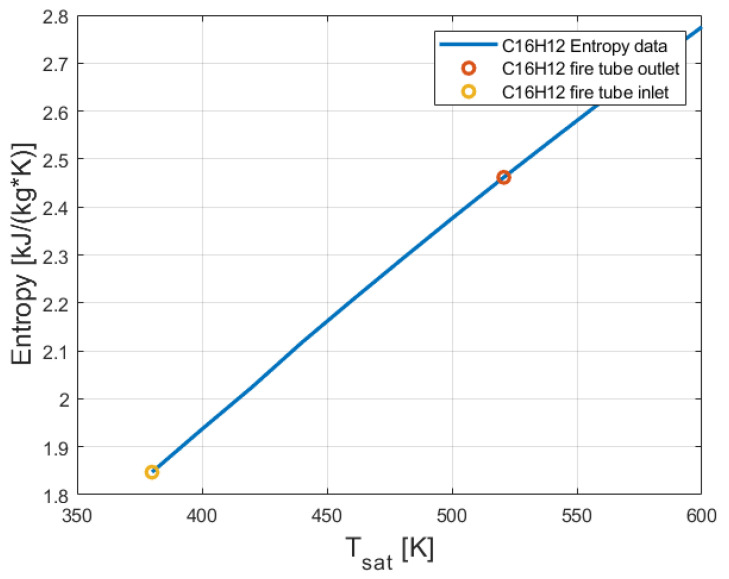
Entropy of 2-phenylnaphthalene (C_16_H_12_) at the inlet and the outlet of the fire tube.

The entropy of 2-phenylnaphthalene at outlet of the fire tube (see Figure 1) has been calculated by performing interpolation between the enthalpy obtained in Equation (4) and the enthalpies shown in [21]. The entropy of 2-phenylnaphthalene at the inlet and outlet of the fire tube is presented in Figure 5.

**Figure 6:**
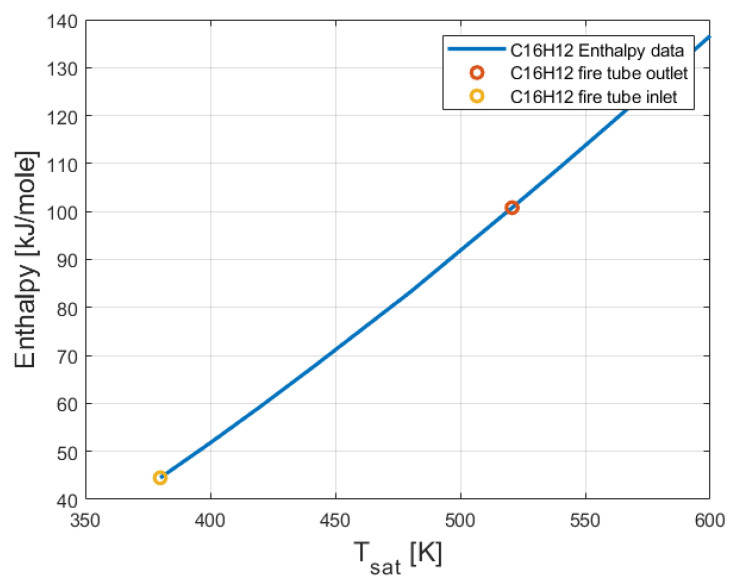
Enthalpy of 2-phenylnaphthalene (C_16_H_12_) at the inlet and the outlet of the fire tube.

From Figure 5, it can be seen that the entropy of 2-phenylnaphthalene (C_16_H_12_) increased with the temperature. The enthalpy of 2-phenylnaphthalene (C_16_H_12_) at the inlet and the outlet of the fire tube is presented in Figure 6.

**Figure 7:**
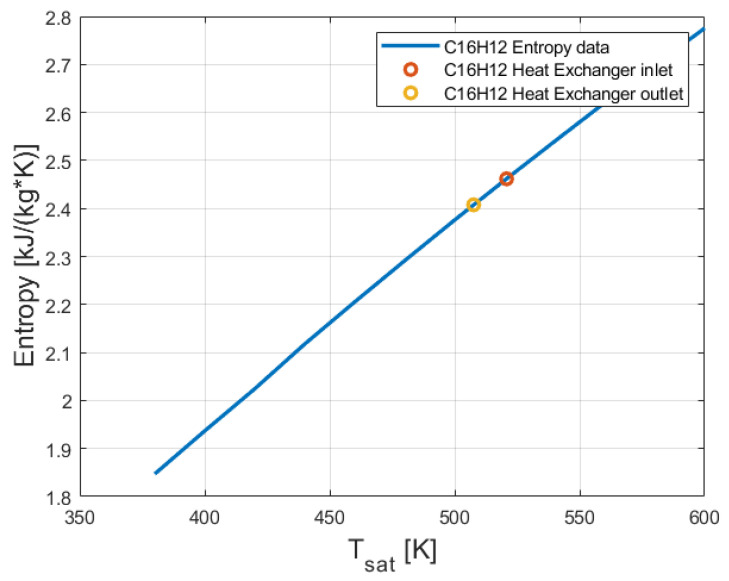
Entropy of 2-phenylnaphthalene (C_16_H_12_) at the inlet and the outlet of the evaporator.

From Figure 6, it can be seen that the enthalpy of 2-phenylnaphthalene (C_16_H_12_) increased with the temperature. The enthalpy and the entropy of the 2-phenylnaphthalene at the outlet of the evaporator (see Figure 1) has been calculated by performing interpolation between the temperature obtained in Equation (5) and the temperatures shown in [21]. The entropy of 2-phenylnaphthalene at the inlet and the outlet of the evaporator is presented in Figure 7.

As expected, the temperature and entropy of 2-phenylnaphthalene decreased. This is because of the heat removed from the 2-phenylnaphthalene stream inside the evaporator. Butane absorbed this heat. Figure 5, Figure 6 and Figure 7 show that the maximal temperatures of 2-phenylnaphthalene were much lower than its boiling temperature (600 K).

## 3. Results

The results section contains two parts. The first part contains the FDS numerical results. The thermodynamic analysis results are presented in the second part.

### 3.1. FDS Results for the Burner

**Figure 8:**
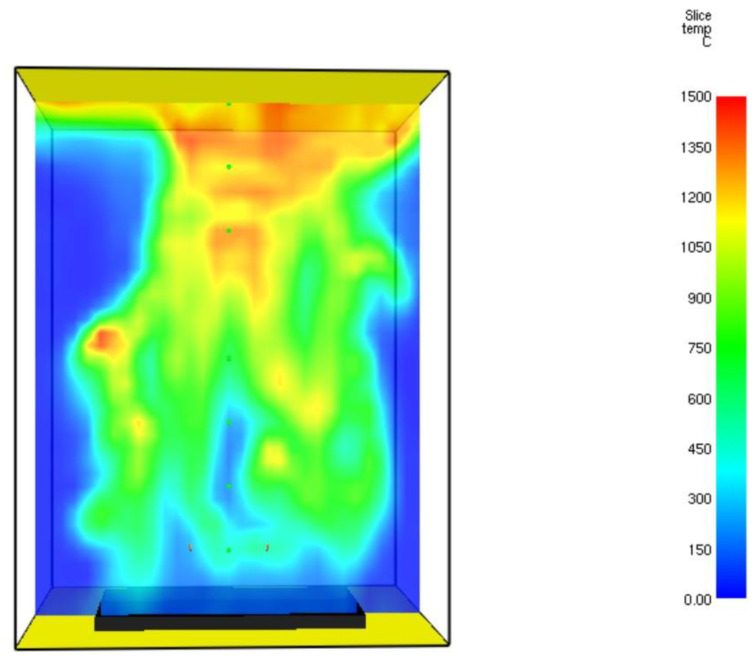
Temperature distribution profile (°C) of the flue gases inside the petroleum coke burner at *t* = 60.7 s.

The numerical model consists of 24,000 numerical cells. The gaseous temperature obtained by the CFD calculation was validated. The temperature distribution profile of the flue gases at *t* = 60.7 s is shown in Figure 8.

**Figure 9:**
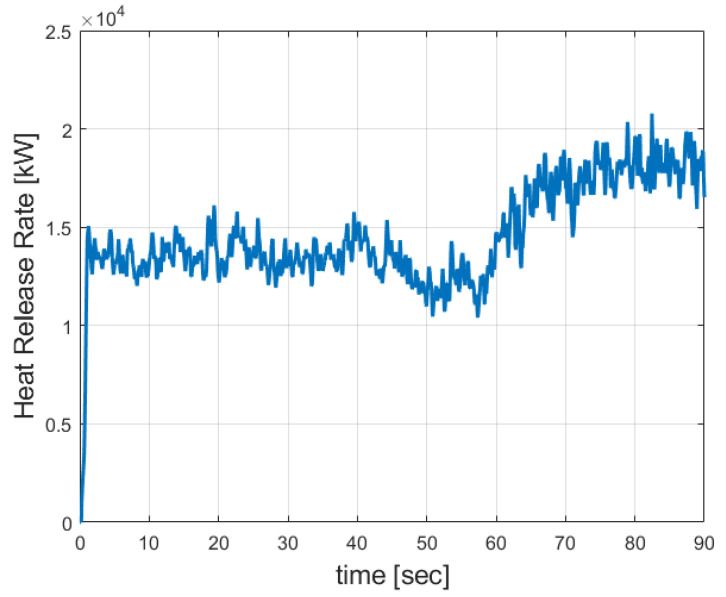
Heat Release Rate (HRR) of the petroleum coke particles.

It can be seen from this figure that the maximal temperature reached was 1440 °C. The hot flue gaseous mixture tended to concentrate in the upper part of the burner. This is because of the buoyancy forces (natural convection). Similar values have been reported in [18]. Figure 9 shows the Heat Release Rate (HRR).

**Figure 10:**
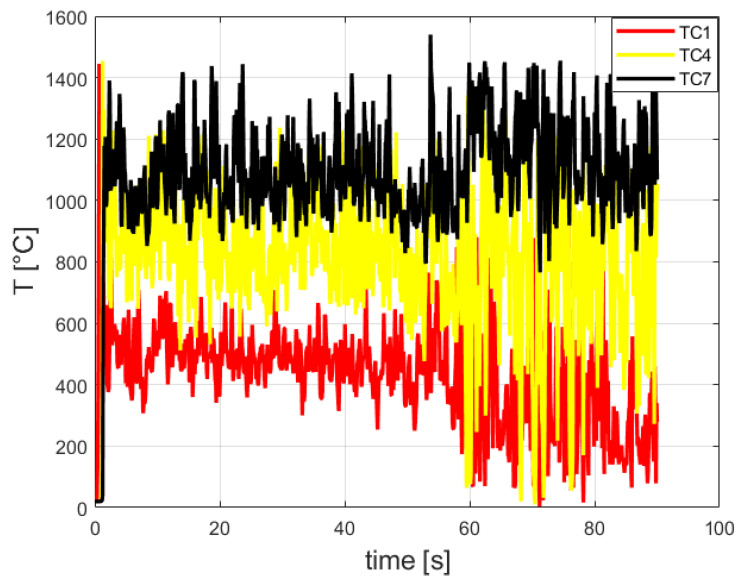
Calculated temperature of three thermocouples inside the burner.

It can be seen that from Figure 8 that the maximal Heat Release Rate was about 20,000 [kW]. Figure 10 provides the temperature readings of the three thermocouples (TC1, TC4, and TC7).

**Figure 11:**
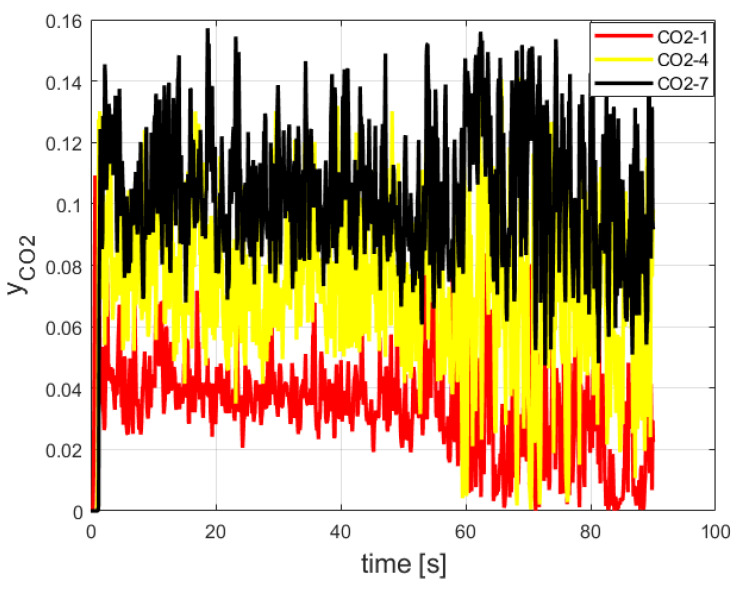
Carbon dioxide readings of three sensors inside the burner.

The carbon dioxide mole fraction readings (CO2-1, CO2-4, and CO2-7) are shown in Figure 11.

According to Figure 10, the maximal CO_2_ mole fraction reading was 15.0%. It is in good agreement with the CO_2_ mole fractions obtained in [22,23]: 14.5% and 13.6%, respectively.

### 3.2. Grid Sensitivity Study Results

**Figure 12:**
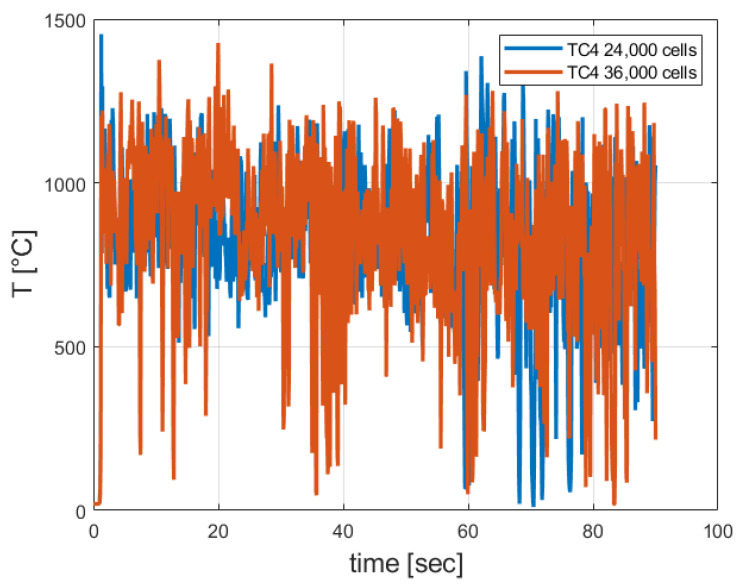
Calculated temperatures obtained by using the two grids.

An additional FDS model containing 36,000 cells was developed. Numerical integration of the instantaneous temperature readings over time was performed in order to calculate the average temperature. The maximal relative error obtained was about 7.0% (see Figure 12).

As can be seen from Figure 12, the calculated temperatures obtained by using the two grids were similar.

### 3.3. Thermodynamic Analysis Results of the Organic Rankine Cycle (ORC)

The enthalpies and entropies of butane calculated at different points of the ORC are summarized in Table 2 (see Figure 1). The isentropic efficiencies of the turbine (expander) and the pump were 0.96 and 0.80, respectively.

**Figure 13:**
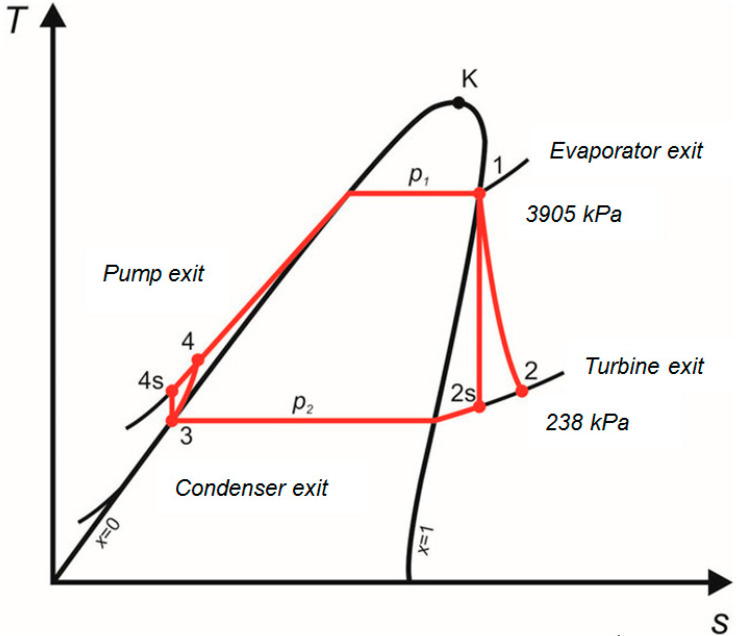
Temperature entropy diagram of the proposed organic Rankine cycle.

The T-s (Temperature entropy diagram) of the ORC cycle is shown in Figure 13.

The calculated heat rates and power values are shown in Table 3. The mass flow rate of butane is 30.44 [kg/s] [24]. It is regarded as one of the best pure fluids in term of its exergy efficiency [24]. It has a low global warming potential (about 4) and zero ozone depletion potential [25]. It has low specific radiative forcing (RF) compared with ethane and propane [26]. However, it is flammable, highly stable and non-corrosive [26]. Since it is a non-corrosive fluid [27,28], major tube failures, such as stress corrosion cracking, may be avoided. According to Bao [29], the maximum net power can be obtained by using butane or pentane working fluids.

According to Table 3, the net power produced by the ORC (the sum of the turbine and pump powers) is equal to the net heat rate (the sum of the evaporator and condenser heat rates). The calculated net power is in good agreement with the net power reported in [24], which is also close to 3472 kW. The relative error is 6.1%. The thermal efficiency is:

$$\eta_{th}=\frac{{\dot{W}}_{net}}{{\dot{Q}}_{boiler}}=\frac{3260}{18072}=0.18=18\%$$

The entropy change rate and irreversibility of each component (turbine, boiler, evaporator, condenser and pump) of the ORC are shown in Table 4:

According to Table 4, the entropy change rates are positive. The entropy change rates of the fire tube and evaporator are much higher than the entropy change rates of the turbine, pump, and condenser. This is because there are sharp temperature gradients between the two heat carriers in these components. A new bioeconomic indicator was applied in this work to evaluate the sustainability of power cycles [30,31]. This allowed us to compare the power cycles (such as Rankine and Brayton) in relation to their sustainability. This indicator decreases with an increasing thermal efficiency. It is defined by the total irreversibility divided by the net power:

$$I=\frac{T_{0}{\dot{S}}_{g}}{{\dot{W}}_{net}}=\frac{22.573}{3.260}≅7.2$$

The indicator of the proposed ORC design is 7.2. It is similar to the indicator of the standard Brayton cycle [30].

## 4. Conclusions

ORC systems apply organic fluids instead of water in order to generate electricity. Because of their lower boiling points and higher vapor pressures compared to water, lower-temperature heat sources can be utilized in order to generate electricity more efficiently. A Large Eddy Simulation (LES) and thermodynamic study was performed on Organic Rankine Cycle (ORC) components (boiler, evaporator, turbine, pump, and condenser). Fire Dynamics Simulator software (FDS) was applied in order to simulate the petroleum burner and to calculate the heat release rate. A grid sensitivity study was carried out. A FDS model containing 36,000 cells was developed. The relative error obtained was about 7.0%. The maximal flame temperature reached was 1440 °C. The hot flue gaseous mixture tended to concentrate at the upper part of the burner. This is because of the buoyancy forces (natural convection). The maximal CO_2_ mole fraction is in good agreement with the CO_2_ mole fraction reported in the literature, 14.5%. The maximal Heat Release Rate was about 20,000 [kW].

The proposed ORC utilizes high-boiling-point phenyl-naphthalene liquid. This design makes the high-boiling-liquid concept a safer method (steam explosion hazard may be prevented) for heating the butane stream. These liquids are reliable when operated at atmospheric pressure. The heating temperature can be varied easily. Heavy forgings for pressure vessels and piping are eliminated, and they are compatible with low-cost materials (plain carbon steel and aluminum alloys can be applied) and have no corrosion potential. Butane is considered highly stable and non-corrosive. It is one of the best pure fluids in terms of its exergy efficiency. It has a low global warming potential and zero ozone depletion potential. It has a low specific radiative forcing (RF). It is highly stable, flammable, and non-corrosive. Major tube failures such as stress corrosion cracking may be avoided. The maximum net power is obtained by using butane and pentane. The proposed ORC design described in this work is safer. This is because the flammable butane is separated from the flame produced in the petroleum coke burner. The enthalpies and entropies of butane were calculated by applying THERMOPTIM thermodynamic software. The calculated net power was 3260 kW. It is in good agreement with the net power reported in the literature. The thermal efficiency of the ORC was determined to be 18.0%. This ORC complies with the two laws of thermodynamics. A new bio-economic indicator has been developed recently to evaluate the sustainability of power cycles. This allows us to compare the power cycles in relation to their sustainability. It decreases as the thermal efficiency of the power cycle increases. The indicator of the proposed ORC design is 7.2. It is similar to the indicator of the Standard Brayton Cycle. Future work will address the issue of CO2 emission reduction.

## 5. Discussion and Future Work

**Figure 14:**
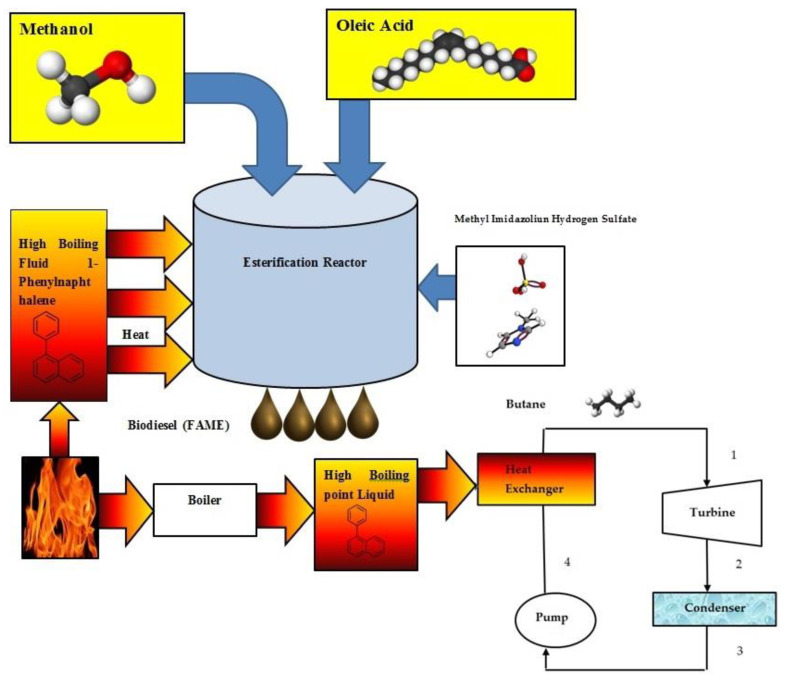
Combined system for producing power and biodiesel (FAME).

The proposed heating system may be utilized for combined biodiesel (FAME) and power production (see Figure 14). Methyl imidazolium hydrogen sulfate ionic liquid is applied as a catalyst of the esterification reaction. The methanol and oleic acid react to form FAME and water in the esterification reactor.

## Figures and Tables

**Table 1:** Thermo-physical properties of 2-phenylnaphthalene [21].

Material Property	Value
*ρ*	335 (kg/m^3^)
*Cp*	2364 (J/(kg·°C))
*k*	0.083 (w/(m·°C))
*η*	0.00011 (Pa·s))

**Table 2:** Enthalpies and entropies at different loactions of the ORC. (the locations are shown in Figure 1).

Point	Pressure [kPa]	Temperature [°C]	Enthalpy, *h* [kJ/kg]	Entropy, *s* [kJ/(kg K)]
*1*	3905	164.2	801.15	2.618
*2*	238	57.5	686.4	2.632
*3*	238	0.0	199.8	0.999
*4*	3905	0.5	207.45	1.004

**Table 3:** Heat and power invested and produced in the ORC system.

Power/Heat	Value [kW]
${\dot{Q}}_{E}$	18,072
${\dot{Q}}_{cond}$	−14,811
${\dot{Q}}_{net}$	3260
${\dot{W}}_{turb}$	3493
${\dot{W}}_{pump}$	−232.3
${\dot{W}}_{net}$	3260

**Table 4:** Entropy change rate and irreversibility of each component of the ORC.

Component	$\dot{S}$ [kW/K]	$T_{0}\dot{S}\;[\mathrm{MW}]$
${\dot{S}}_{turb}$	0.426	0.127
${\dot{S}}_{cond}$	4.515	1.346
${\dot{S}}_{pump}$	0.152	0.045
${\dot{S}}_{fire\_tube}$	46.43	13.800
${\dot{S}}_{E}$	24.34	7.256

## Data Availability

Not applicable.

## Abbreviations

CFD	Computational Fluid Dynamics
CTL	Coal to Liquids
FDS	Fire Dynamics Simulation
F-T	Fischer Tropsch
HRR	Heat Release Rate
SSSF	Steady State Steady Flow
RTE	Radiation Transport Equation

Nomenclature

g	gravity acceleration in [m/s^2^]
s	specific entropy of the stream in [kJ/(kg K)]
t	time in [s]
V	velocity of the entering/leaving stream in [m/s]
Z	height of the stream in [m]
Subscripts	
cond	condenser
c.v	control volume
e	leaving
E	Evaporator
HRR	Heat Release Rate
i	entering
net	net
pump	pump
turb	turbine
Greek letters	
$\eta_{th}$	thermal efficency of the Organic Rankine Cycle
ρ	density in [kg/m^3^]
